# Formulation and assessment of chickpea pulao using fenugreek seeds and Indian rennet to improve blood glycemic levels

**DOI:** 10.1002/fsn3.4107

**Published:** 2024-03-18

**Authors:** Misha Arooj, Zaheer Ahmed, Nauman Khalid, Hafiz A. R. Suleria

**Affiliations:** ^1^ Department of Human Nutrition and Dietetics, School of Food and Agricultural Sciences University of Management and Technology Lahore Pakistan; ^2^ Department of Nutritional Sciences and Environmental Design, Research Complex Allama Iqbal Open University Islamabad Pakistan; ^3^ College of Health Sciences Abu Dhabi University Abu Dhabi United Arab Emirates; ^4^ School of Agriculture, Food and Ecosystem Sciences, Faculty of Sciences The University of Melbourne Parkville Victoria Australia

**Keywords:** chickpea pulao, cuisine modification, hypoglycemic herbs, Pakistani cuisine

## Abstract

Diabetes is becoming a significant health concern in Asia, where the prevalence has reached alarming levels. An important contributing factor is the consumption of high‐carbohydrate foods, including rice, bread, etc. These high‐carbohydrate foods pose a major risk to public health due to their impact on postprandial hyperglycemia. This research aimed to formulate a chickpea pulao (cooked Indian–Pakistani rice dish) and to evaluate its effects on postprandial blood glucose levels in type 2 diabetic individuals. Antioxidant potential and total phenolic contents of herbs at different concentrations (1, 3, 5, 7, and 9%) were measured through DPPH and Folin Ciocalteu assays. The antidiabetic potential was tested by α‐amylase and α‐glucosidase inhibition assays. After sensory evaluation, the best‐chosen concentration was used to formulate the chickpea pulao. The study trial was advertised under “DP trial,” and 12 participants were recruited. A single‐blind randomized cross‐over trial was conducted for 3 weeks with a one‐week wash‐over time in between. Participants' preprandial and postprandial blood glucose levels were recorded for control and intervention recipes. Results indicated that both fenugreek seeds (FS) and Indian rennet (IR) showed good antioxidant and hypoglycemic activity (*p* = .000) in raw and boiled extracts. For DPPH, the IC_50_ values of unboiled and boiled combined (FS + IR) extracts were calculated as 7.4% and 8.02%, respectively. Similarly, for α‐amylase, the IC_50_ values of combined IR and FS unboiled and boiled extracts were 6.58% and 6.83%, and for α‐glucosidase inhibition assay, the values were measured as 14.98% and 16.24%. The single‐blind randomized cross‐over trial showed that consuming the intervention recipe significantly reduced postprandial hyperglycemia (*p* = .000) in type 2 diabetic participants. The intervention recipe decreased hyperglycemia by approximately 15% daily compared to the control recipe. Incorporation of hypoglycemic herbs into dietary patterns can work as an adjunct therapy for diabetes management, especially in populations with a high prevalence of this disease.

## Graphical abstract

Fenugreek seeds and Indian rennet were used to modify chickpea pulao for modulating the blood sugar levels in a single‐blind cross‐over randomized control trial. The results indicate that culinary modification can modulate blood glucose levels.

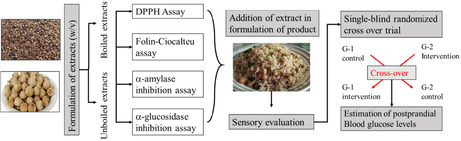

## 1 INTRODUCTION

Diabetes mellitus is a chronic, multifactorial metabolic disorder comprising acute and chronic etiologies, affecting approximately 536.6 million people worldwide (Kleinberger & Pollin, 2015; Lumb, 2014), including people in developed and developing countries. According to IDF (International Diabetes Federation) diabetes atlas, almost half a billion people are living with diabetes worldwide, with a prevalence of 10.5%, which is expected to rise to 12.2% by 2045 (Sun et al., 2022). Type 2 diabetes is rapidly becoming a significant health concern in Asia, particularly in China, India, Pakistan, Bangladesh, Sri Lanka, and Nepal (Hills et al., 2018). In Pakistan, diabetes is at an all‐time high, with the current prevalence rate of 9.6%, which is expected to rise to 15% by 2040. This will take Pakistan to rank fourth among the highest‐ranked diabetes‐affected countries in the world (Bukhsh et al., 2020).

Asian diet generally consists of wheat and rice, both high carbohydrate‐content foods. Pakistani cuisine is rich in flavor and variety, reflecting the country's diverse cultural and ethnic influences. Bread and rice are two of the most important staples of traditional Pakistani meals, which frequently revolve around high‐carbohydrate foods (Rafique et al., 2022). Traditional Pakistani dishes such as biryani, pulao, and naan bread are typically high in carbohydrates, unhealthy fats, and sugars, majorly contributing to the obesity epidemic and insulin resistance, the two culprits and underlying causes of type 2 diabetes. In Pakistan, the food is generally cooked in an enormous amount of vegetable oils, partially hydrogenated, and animal fats. Mostly, animal protein is consumed with saturated fat, and the food is generally cooked on high heat; this kills the food's beneficial bioactives and nutrients, including heat‐sensitive vitamins, various antioxidants, and some flavonoids (Lee et al., 2018).

Herbal therapy alone or in combination can be a significant factor in managing diabetes. Though sometimes misinterpreted as misleading and unscientific, herbal medicines show better synergistic effects than others. For example, Metformin, the first line of defense against type 2 diabetes, is also made from *Galega officinalis* or *French lilac* (Chang et al., 2013).

Fenugreek (*Trigonella foenum‐graecum*) and Indian rennet (*Withania coagulans*) are two herbs that have been traditionally used in Indian and Middle Eastern cuisines and medicinal practices for their health benefits, including hypoglycemic effects. Fenugreek seeds are tiny, golden‐brown seeds with a maple‐like flavor and bitter taste, and are highly valued for their various health advantages, flavor, and aroma. Volatile oils and alkaloids are the two major bioactive compounds responsible for an unpleasant odor and a bitter taste. Fenugreek seeds are rich in iron, magnesium, and other trace minerals, and also contain saponins and alkaloids that improve insulin sensitivity, increase glucose uptake, and reduce glucose production in the hepatic system (Geberemeskel et al., 2019).

Proximate analysis showed that fenugreek seeds contain significant fiber (5–60 g/100 g). Additionally, it aids in controlling blood sugar levels and reducing glucose absorption into the bloodstream, thus improving insulin's action. Galactomannans are most of the soluble fibers found in seeds responsible for lowering the body's glucose absorption (Meghwal & Goswami, 2012). In addition, fenugreek endosperm also contains 35% alkaloids, mostly trigonelline (Jani et al., 2009). All these substances are categorized as biologically active because they exert notable pharmacological effects on the human body. Furthermore, because they have hypoglycemic, antilipidemic, anti‐carcinogenic, and cholagogic qualities, adding these seeds to daily diets helps to treat hypercholesterolemia, cancer, and diabetes mellitus (Meghwal & Goswami, 2012). Literature suggests that oral intake of fenugreek seeds reduces triglycerides by lowering low‐density lipoproteins and boosting high‐density lipoproteins (Ahmad et al., 2016).

Indian rennet herb, *Withania coagulans*, or milk‐clotting herb, is a plant‐based coagulant, native to the arid regions of India, Pakistan, and Afghanistan and a nightshade family member, sometimes referred to as “vegetable rennet” or “Indian cheese maker” since its fruits and leaves are used to coagulate milk. Indian rennet fruits' ability to cause milk to coagulate is linked to an enzyme called withanin in the pulp and husk (Khan et al., 2021).

The phytochemical analysis of the aqueous and ethanolic extract of *Withania coagulans* revealed a variety of different phytonutrients, including steroidal lactones, alkaloids, flavonoids, tannin, saponins, organic acids, withanolides, withaferin A, etc. that are responsible for hypoglycemia activity of the flowers (Agarwal et al., 2014). *Withania coagulans* have been used for centuries in traditional medicine to lower blood sugar levels and improve insulin sensitivity. Fruit of Indian rennet flowers are known to possess antidiabetic properties and are said to be because of magnesium and calcium (Khan et al., 2021).

These herbs can be added to daily diets to cater to alarmingly high risk of non‐communicable diseases. Limited studies are available worldwide that measure the effect of these local herbs in modifying cuisine, but the evidence is certainly lacking in Pakistani cuisine modification. The present study evaluated the phenolic and antioxidant profile of diabetes‐modulating herbs, the hypoglycemic effect of selected herbs in chickpea pulao, and its impact on postprandial blood sugar levels in type 2 diabetic individuals.

## 2 MATERIALS AND METHODS

### 2.1 Chemicals and materials

The Indian rennet/paneer dodi flowers and fenugreek seeds were purchased from the local market and brought to the laboratory for thorough cleaning and decontamination from dust particles or stones. The chemicals used for analysis were of analytical grade and were used as such.

### 2.2 Preparation of extracts

Aqueous extracts of whole Indian rennet, fenugreek seeds, and combined extracts were formulated using distilled water with varying concentrations from 1% to 9% (w/v) that were chosen based on sensory evaluation of aqueous herb extracts. Since these extracts were used as the broth medium to cook rice, the bitterness of Indian rennet and the nutty flavor of fenugreek seeds were judged acceptable at 9% (w/v). After soaking herbs for 24 h, the extracts were strained and centrifuged at 550g for 5 min. The supernatant was separated, and the remaining extract was stored in the refrigerator at 4°C for further usage.

Since the extracts were supposed to be used in the broth while making pulao to analyze the impact of cooking on herb extracts and to measure the efficacy of plant bioactives, all extracts of different sources and concentrations were boiled at 100°C for 15 min to compare any change in the antioxidant and antidiabetic potential of selected herbs after boiling.

### 2.3 Determination of total phenolic contents

To quantify the total phenolic contents (TPC) in plant extracts, the Folin–Ciocalteu method based on an oxidation–reduction reaction was performed (Niroula et al., 2019). Briefly, 0.5 mL of each sample extract was added in falcon tubes along with 2.5 mL of freshly prepared Folin–ciocalteu reagent, and 2.5 mL of 7.5% sodium carbonate was added after 5 min intervals and incubated at 45°C for 45 min. The absorbance was measured at 765 nm against the blank solution. Various concentrations of gallic acid were used as standard, and the results of TPC were presented as mg GAE/100 g.

### 2.4 Determination of total antioxidant capacity

The free radicals’ scavenging properties of aqueous extracts at different concentrations were tested using DPPH assay (Verma et al., 2017). Briefly, 25 μL of each sample extract was mixed with 250 μL Tris–HCl base and 1 mL of DPPH reagent in foiled falcon tubes and were thoroughly mixed. The foiled falcon tubes were then kept in the dark for 30 min. The absorbance was measured at *t* = 0 min and *t* = 30 min at 517 nm. The free radical scavenging activity of herb extracts was calculated as Equation 1.

(1) $$\text{Scavenging activity}\;\left(\%\text{inhibition}\right)=100-\left[\left(\frac{At30}{Ato}\right)\times 100\right].$$

At_30_ is the absorbance at *t* = 30, while At_0_ is the absorbance at *t* = 0.

### 2.5 Α‐amylase inhibition assay

α‐amylase inhibitory activity of the extract was performed according to this method with minor modification (Telagari & Hullatti, 2015). Briefly, 50 μL of 100 mM phosphate buffer (pH = 6.8), 10 μL α–amylase solution (2 U/mL), and 20 μL of various concentrations of the extract were added in eppendorfs and preincubated at 37°C for 20 min. After preincubation, 20 μL of 1% soluble starch was added as the substrate, and the solution was again incubated further at 37°C for 30 min. After 30 min, 100 μL DNS color reagent was added and was further boiled for 10 min. The absorbance was measured at 540 nm. The results were expressed as percentage inhibition, which was calculated using the formula,

(2) $$\;\text{Inhibitory activity}\;\left(\%\right)=\left(1-\frac{\mathrm{As}}{\mathrm{Ac}}\right)\;x\;100,$$

where As is the absorbance of the sample and Ac is the absorbance of the control.

### 2.6 Α‐glucosidase inhibition assay

α‐glucosidase inhibitory activity of the extract was performed according to Telagari and Hullatti (2015) with minor modification. In Eppendorfs, 50 μL of 100 Mm phosphate buffer with pH = 6.8, 10 μL of α‐glucosidase solution (1 U/mL), and 20 μL of different concentrations of the extract were added and preincubated at 37°C for 15 min. After 15 min, 20 μL of freshly prepared 5 mM *p*‐NPG solution was added as a substrate and incubated further at 37°C for 20 min, and the reaction was stopped by adding 50 μL of 0.1 M Na_2_CO_3_. The absorbance of the released *p*‐nitrophenol was measured at 405 nm. The results were presented as percentage inhibition, calculated using Equation 3.

(3) $$\text{Inhibitory activity}\;\left(\%\right)=\left(1-\frac{\mathrm{As}}{\mathrm{Ac}}\right)x\;100,$$

where As is the absorbance of the sample and Ac is the absorbance of the control.

### 2.7 Value addition in chickpea pulao

**FIGURE 1:**
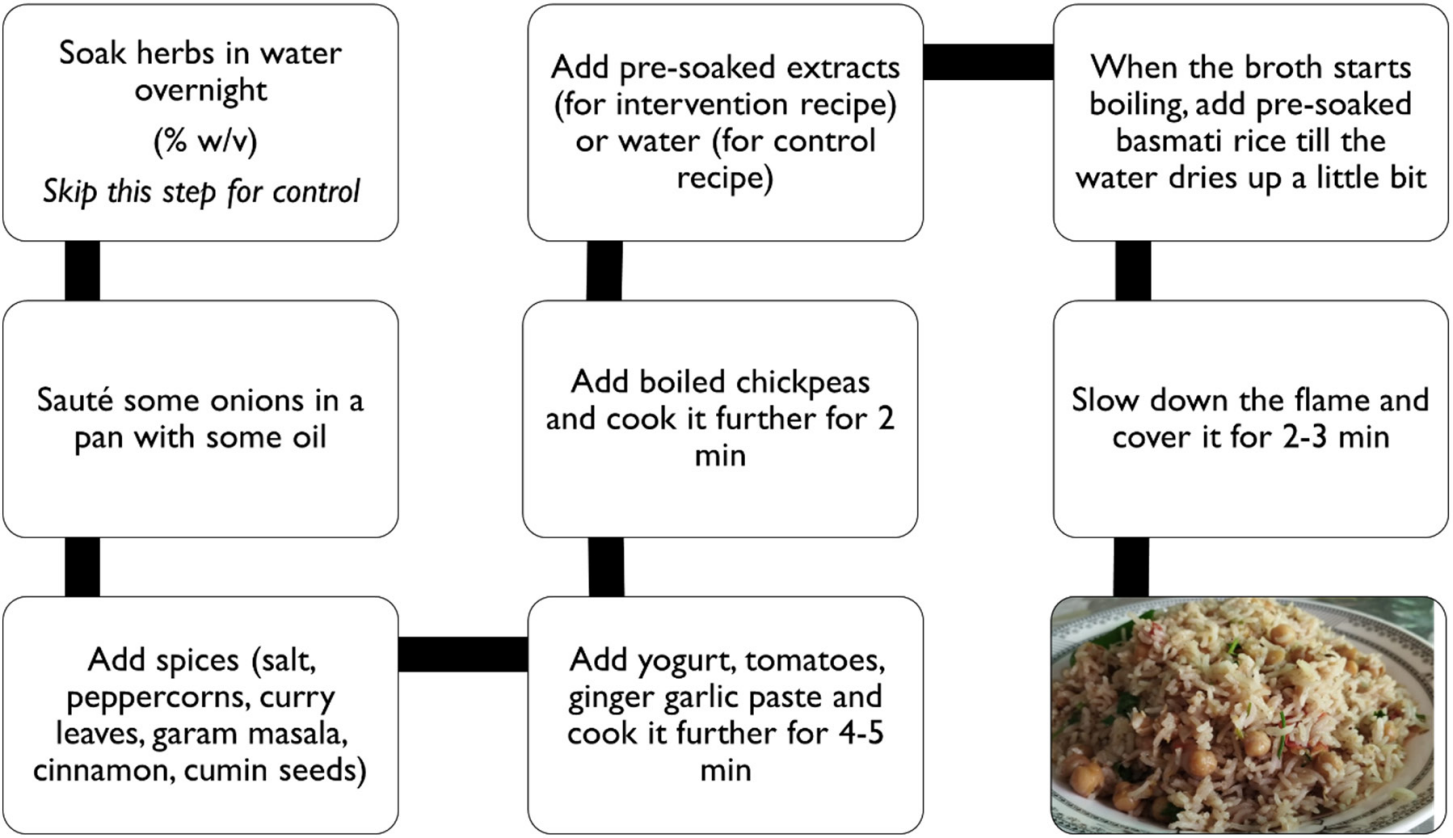
The detailed procedure for developing chickpea pulao for control and intervention recipes.

Table 1 shows the different treatments used for the formulations of chickpea pulao. Water was used as the broth medium for the controlled recipe (Figure 1), whereas the treatment recipes were cooked in overnight presoaked herbs extracts.

**TABLE 1:** Treatment plan for the formulation of antidiabetic pulao.

Treatments	FS (g)	IR (g)	Broth (mL)
T_o_	0	0	100
T_1_	1	1	98
T_2_	3	3	94
T_3_	5	5	90
T_4_	7	7	86
T_5_	9	9	82

### 2.8 Sensory evaluation of chickpea pulao

A trained panel of 10 judges was formed based on sensitivity testing, and a group of 10 individuals was also selected as the general population to assess the overall acceptability of the product. Before evaluation, all participants were briefed about this study and its treatments. All 20 participants were given treatments made with different herb extract concentrations and were coded randomly through preassigned codes to limit any biases in scoring. All participants were given a questionnaire to number their sensory observations based on the 9‐point hedonic scale, and results were analyzed through the Kruskal–Wallis test.

### 2.9 Recruitment of participants

**FIGURE 2:**
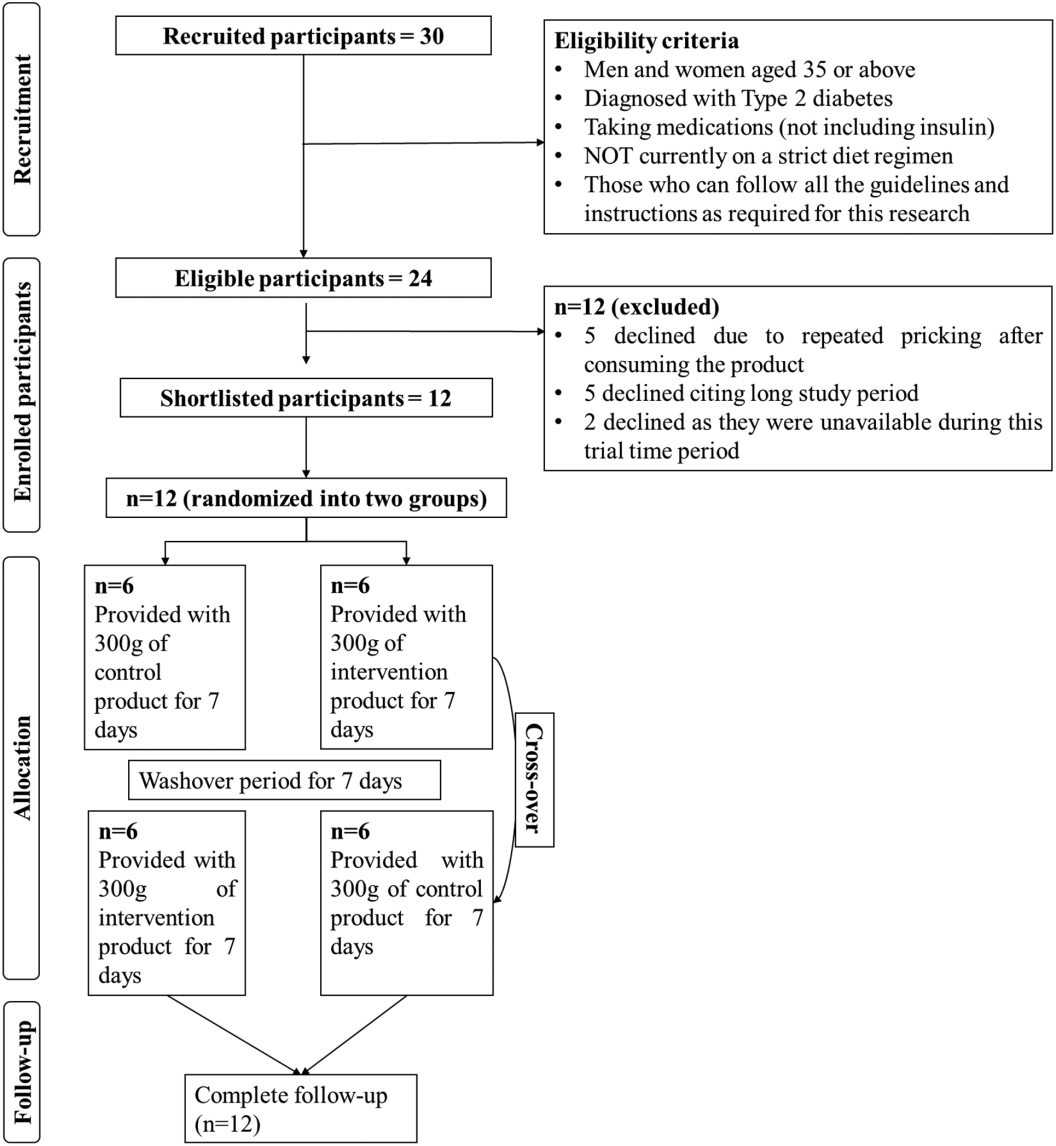
PRISMA for recruitment of participants for DP trial.

The trial was advertised under the “DP trial” name for recruiting participants. A total of 30 participants were recruited for the trial. However, out of 30 (Figure 2), only 24 met the inclusion criteria. Upon providing all the details regarding the DP trial, 12 opted out of the study. Out of 12, 5 participants opted out, citing repeated pricking as the primary cause of discontinuation of the research trial, and five declined due to the longer study duration. While two declined due to unavailability during the trial period. After the recruitment process, all participants were called upon for the interview and discussion session. They were asked to complete a questionnaire based on their demographic history, family history of disease, and dietary history. Every participant was explained about the study trial, dose, and duration.

### 2.10 Study design

A single‐blind randomized cross‐over design was used. Briefly, 12 eligible participants were randomized into two groups, i.e., G‐1 and G‐2, with three males and three females in each group. Participants were provided with 300 g of the control and intervention recipe. Preprandial blood glucose levels were recorded before providing the control and intervention recipe. After consuming the product, postprandial glucose levels were measured after 1 and 2 h using a digital glucometer. The type of product, whether intervention or control, was not revealed to the participants to eliminate any risk of biases. The trial continued for twenty‐one days.

### 2.11 Ethical approval statement

The ethical approval to conduct this study was obtained from the bioresearch ethical committee of the University of Management and Technology Lahore via. Approval number UMT/IRB/PostGrad/Res/2022‐03‐R002‐C‐1. The clinical trial was listed on ClinicalTrial.gov (NCT06095622). Moreover, the study was conducted in accordance with the principles of the Declaration of Helsinki Protocol.

### 2.12 Statistical analysis

All experiments were done on unboiled and boiled extracts and in triplicate. Differences in mean values were tested using a one‐way analysis of variance, and post hoc analysis was done using Tukey's test. Comparison between unboiled and boiled extracts was determined through paired sample *t*‐test using SPSS 25, and differences were considered significant at 99% probability *(p* < .01). The results were expressed as mean ± standard deviation.

## 3 RESULTS AND DISCUSSION

### 3.1 Antioxidant potential of sample extracts

**FIGURE 3:**
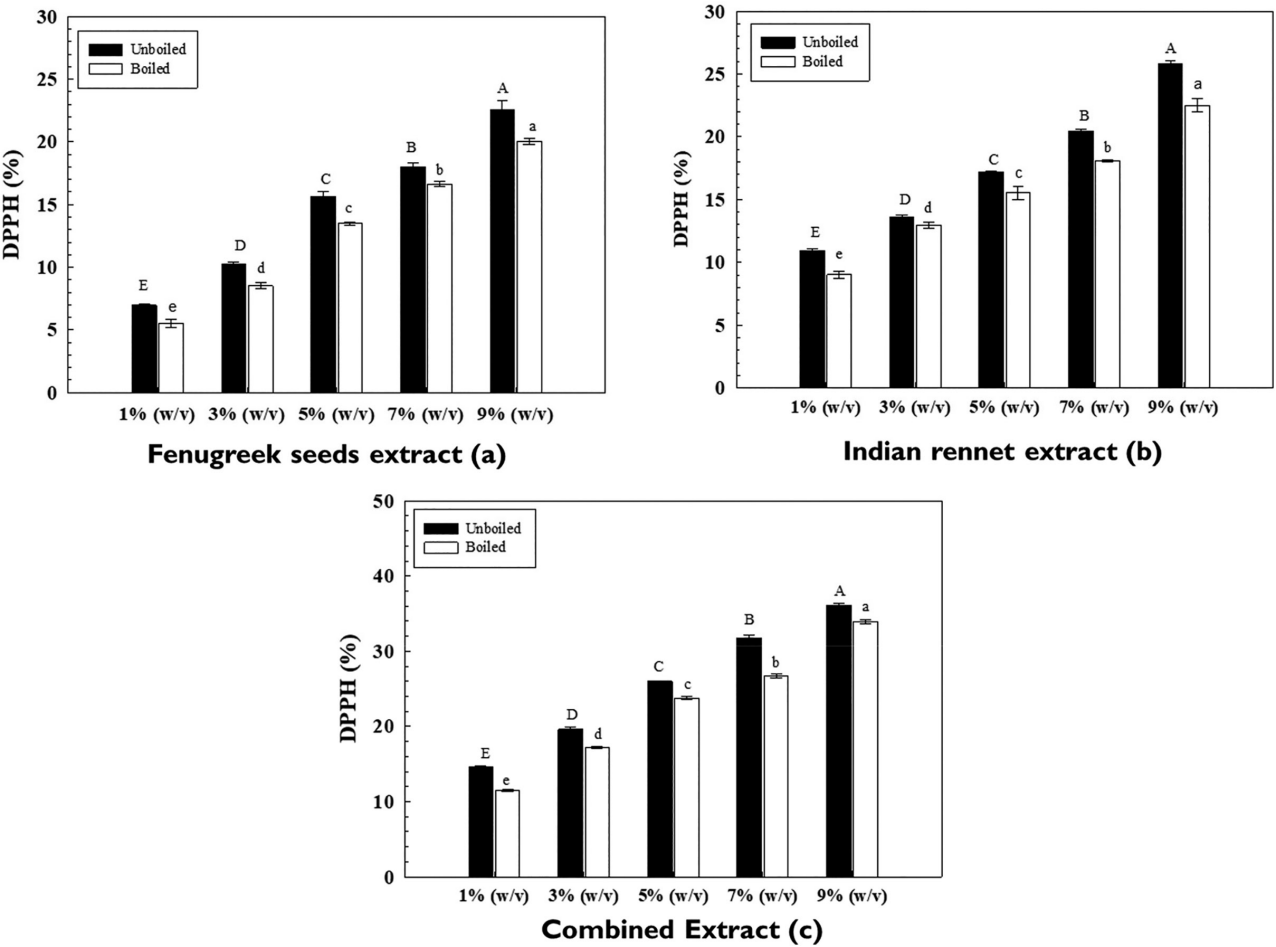
DPPH activity of extracts. Uppercase and lowercase letters show significant differences between unboiled and boiled extracts.

Medicinal plants such as fenugreek and Indian rennet have been attributed to possess multipotent free radical scavenging antioxidant activities due to naturally occurring polyphenols and phytochemicals. The mean free radical scavenging activity of both unboiled and boiled fenugreek seeds and Indian rennet extracts varied significantly (*p* = .000) with varying concentrations. DPPH activity of both boiled and unboiled extracts increased (Figure 3a) with the increase in the concentrations linearly. As a result, the IC_50_ value of unboiled FS extract was 12.1%, while the IC_50_ value for boiled FS was 12.7%. Whereas a similar trend was observed (Figure 3b) for Indian rennet (IR) extracts, for unboiled extracts, the highest activity was measured in 9% (w/v) extract (25.793 ± 0.30%), and relatively lower activity was measured in 1% (w/v) extract. Meanwhile, the boiled extracts also exhibited a similar trend. The IC_50_ value of unboiled and boiled Indian rennet extract was 11.8% and 13.7%, respectively. IR's free radical scavenging activity is mainly attributed to the presence of withanolides (Sun et al., 2016).

The additive impact (Figure 3c) of both FS and IR extract was also tested against the DPPH activity. The result depicted that those combined extracts significantly increased the synergistic impact of the extracts’ free radical scavenging activity. The IC_50_ values of unboiled and boiled combined (FS + IR) extracts were calculated as 7.4% and 8.02%, respectively. Temperature has a significant impact on the antioxidant activity of herbs and vegetables. Boiling of both FS and IR extracts showed a significant decrease in antioxidant levels. This is mainly because many antioxidants are water soluble, and leaching out during boiling or steaming results in antioxidant loss (Kinyi et al., 2022).

Various studies have analyzed the antioxidant potential of FS and IR extracts. Kumar et al. (2021) reported that the DPPH activity (IC_50_) of aqueous FS extract was measured as 62 mg/mL. Similarly, another study reported that the DPPH (IC_50_ μg/mL) activity of FS in water as the solvent was found to be 66.6 μg/mL whereas, in methanol, a relatively lower DPPH activity was measured, i.e., 56.4 μg/mL (Hameed et al., 2019). In another study, the free radical scavenging activity of water extracts was found to be 10.31% at 0.5 mg/mL concentration, whereas the hot water FS powder extract showed inhibition activity of 50.8% at 0.45 mg/mL concentration (Norziah et al., 2015).

Several studies have also documented DPPH activity of IR. Nishtha et al. (2017) tested different concentrations of aqueous extract of *Withania coagulans* ranging from 0 to 200 μg/mL. Results showed that as the concentration increased, the DPPH scavenging activity of IR extract increased, and the higher concentration resulted in the scavenging activity of 73.9% at 200 μg/mL. Moreover, the IC_50_ value was measured as 69 μg/mL. Hemalatha et al. (2004) tested the DPPH activity of aqueous Indian rennet extracts at 2 mg/mL concentration. The results concluded that the scavenging activity of the aqueous extract was 605.1 μL. It is known that natural phytochemicals and flavonoids present in herbs are responsible for antioxidant activity. All available studies showed a similar trend regarding the DPPH activity of fenugreek seed extracts (Wijekoon et al., 2011).

### 3.2 Determination of total phenolic content of herb extracts

**FIGURE 4:**
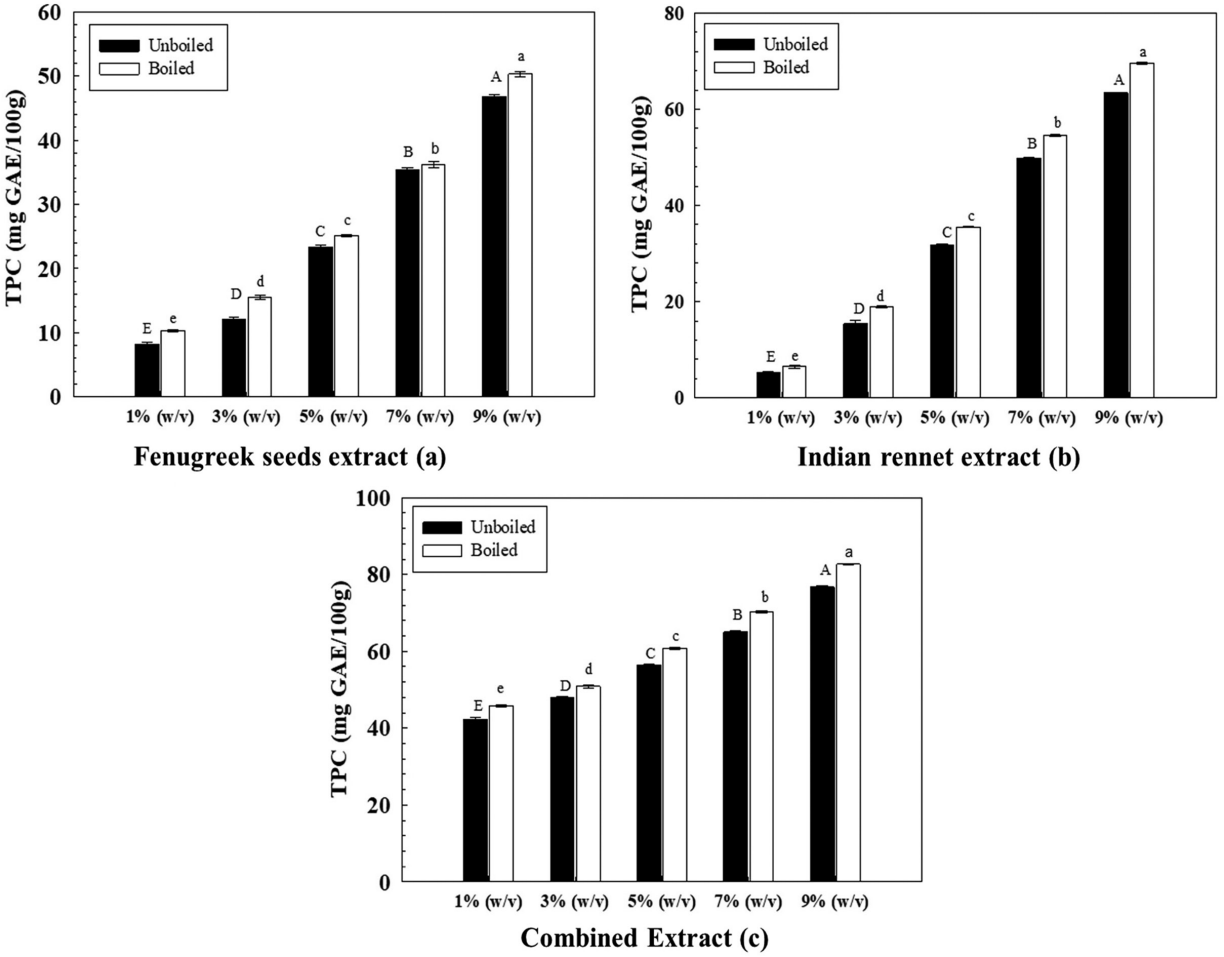
Total phenolic content of extracts. Uppercase and lowercase letters show significant differences between unboiled and boiled extracts.

Phenolic compounds are significant plant nutrients with prominent redox properties. In addition, the hydroxyl group attached to some plant constituents exhibits free radical scavenging activity. For the unboiled aqueous FS extracts, the highest phenolic content was found in 9% (w/v), i.e., 46.9 ± 0.3 mg GAE/100 g, and the lowest content was measured in 1% (w/v). The TPC (Figure 4a) for boiled FS extracts ranged from 10.3 ± 0.2 to 50.3 ± 0.5 mg GAE/100 g and followed a linear relationship. Whereas for unboiled IR extracts (Figure 4b), the TPC ranged from 5.3 ± 0.1 to 63.4 ± 0.1 mg GAE/100 g, whereas for boiled IR extracts, the TPC varied from 6.5 ± 0.3 to 69.6 ± 0.3 mg GAE/100 g.

For combined extracts, higher concentrations yielded more phenolic content than lower concentrations. In addition, the correlation further reaffirmed a linear relationship (Figure 4c) between concentrations and total phenolic compounds. Boiling or heating herbs results in nutrient loss. Although some polyphenols are highly heat‐labile, few phenolic compounds can survive higher temperatures (Maghsoudlou et al., 2019).

Dixit et al. (2005) reported that with water extraction of ground FS 1:20 solids to water, the TPC was measured to be 10 mg GAE/g. Similarly, Hameed et al. (2019) characterized the total phenolic content of fenugreek seeds in both methanol and water as solvents. Results reported that the TPC of FS was found to be 241.5 mg GAE/100 g in an aqueous solvent. Another study also reported lower TPC in water‐based extraction methods than in organic solvents such as acetone, ethanol, and methanol. The primary reason is that many phytochemicals are generally organic compounds better dissolved in organic solvents (Mashkor, 2014).

Similarly, for Indian rennet extract, a study by Nishtha et al. (2017) reported that the total phenolic content measured in the aqueous extract was 15.6 mg GAE/mL when compared with gallic acid as the standard. Another study was conducted in which different sources of IR roots were tested for TPC. The phytochemical investigation measured the range of TPC of IR root extracts to be 14.91–23.7μgGAE/mg DW (Valizadeh et al., 2015). The difference in polyphenolic yield from these study findings is attributed to the difference in the solvent‐to‐sample ratio, time of the experiment, and extraction method (Kowalczyk et al., 2013).

### 3.3 Α‐amylase inhibition potential of herb extracts

Medicinal plants used in the traditional treatment possess antidiabetic constituents, mainly characterized as α‐amylase inhibitors. This study used the DNS method to analyze the selected herbs for α‐amylase inhibition activity.

**FIGURE 5:**
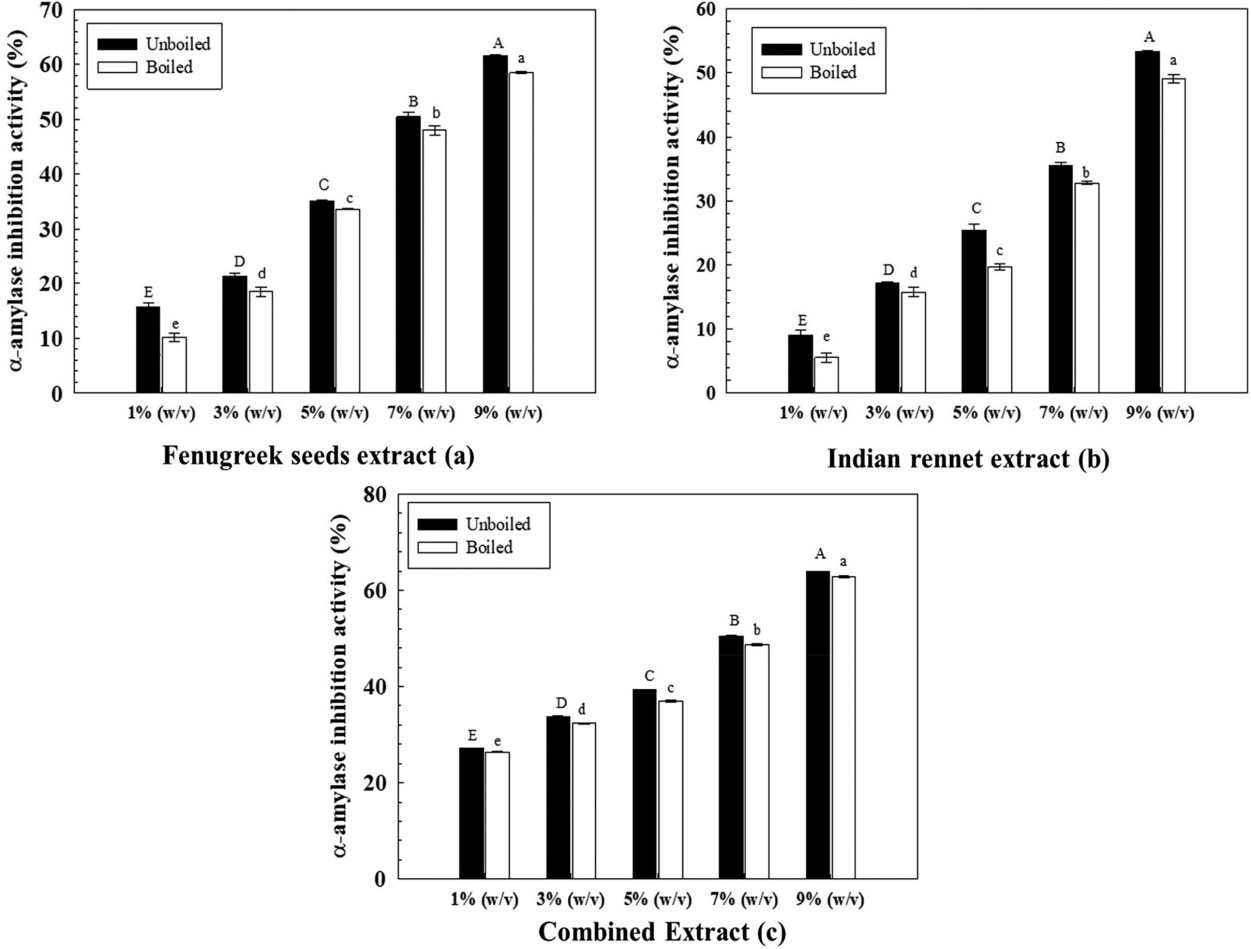
α‐amylase inhibition activity of extracts. Uppercase and lowercase letters show significant differences between unboiled and boiled extracts.

About 1% (w/v) unboiled FS extract showed the lowest α‐amylase inhibition activity, i.e., 15.7% and highest in 9% extract (61.6%). The same trend was followed by boiled FS extract (Figure 5a) as α‐amylase inhibition activity ranged from 10.1% for 1% (w/v) to 58.4% for 9% (w/v). The IC_50_ values of unboiled and boiled fenugreek extracts were recorded as 7.17% and 7.57%, respectively. Along with other polyphenols and flavonoids, α‐amylase activity in fenugreek seeds is attributed to a plant hormone called Trigonelline, which is known to have hypoglycemic activity by reducing the risk of diabetic neuropathy and hyperlipidemia (Naika et al., 2022). On the other hand, a rather similar trend (Figure 5b) was observed for IR extracts. The 9% (w/v) showed the most significant inhibitory activity (53.3%). The IC_50_ value of unboiled and boiled IR extracts was 9.09% and 9.31%. The combined extracts (Figure 5c) showed additive impact. The IC_50_ values of combined IR and FS unboiled and boiled extracts were 6.58% and 6.83%, respectively.

Prithiksha et al. (2022) tested the α‐amylase activity of 80% aqueous fenugreek seed extract at different concentrations ranging from 100 to 500 μg/mL. Results depicted the linear relationship between concentrations and α‐amylase inhibition as the % inhibition activity ranged from 30% at 100 μg/mL to 85% at 500 μg/mL. Sunita (2020) also tested the α‐amylase inhibitory activity of 1 g of fenugreek seed powder extract in 200 mL of water and concluded that the 45.92% α‐amylase inhibitory activity was found in aqueous extract. An in vitro study was conducted for the α‐amylase inhibition activity of Indian rennet extracts (methanol: water, 90:10) at different fractions and concentrations. Results depicted that α‐amylase inhibition was dose‐dependent, and thus with the increase in concentration, the enzyme inhibition activity also increased. However, the literature is limited for the comparison with aqueous extract (Datta et al., 2013).

### 3.4 Α‐glucosidase inhibition activity of extracts

Many phytonutrients in Ayurvedic medicine, such as flavonoids, alkaloids, glycosides, and phenolics, are known to have some α‐glucosidase inhibition tendencies. This study tested the selected herbs against α‐glucosidase activity using the *p*‐NPG method.

**FIGURE 6:**
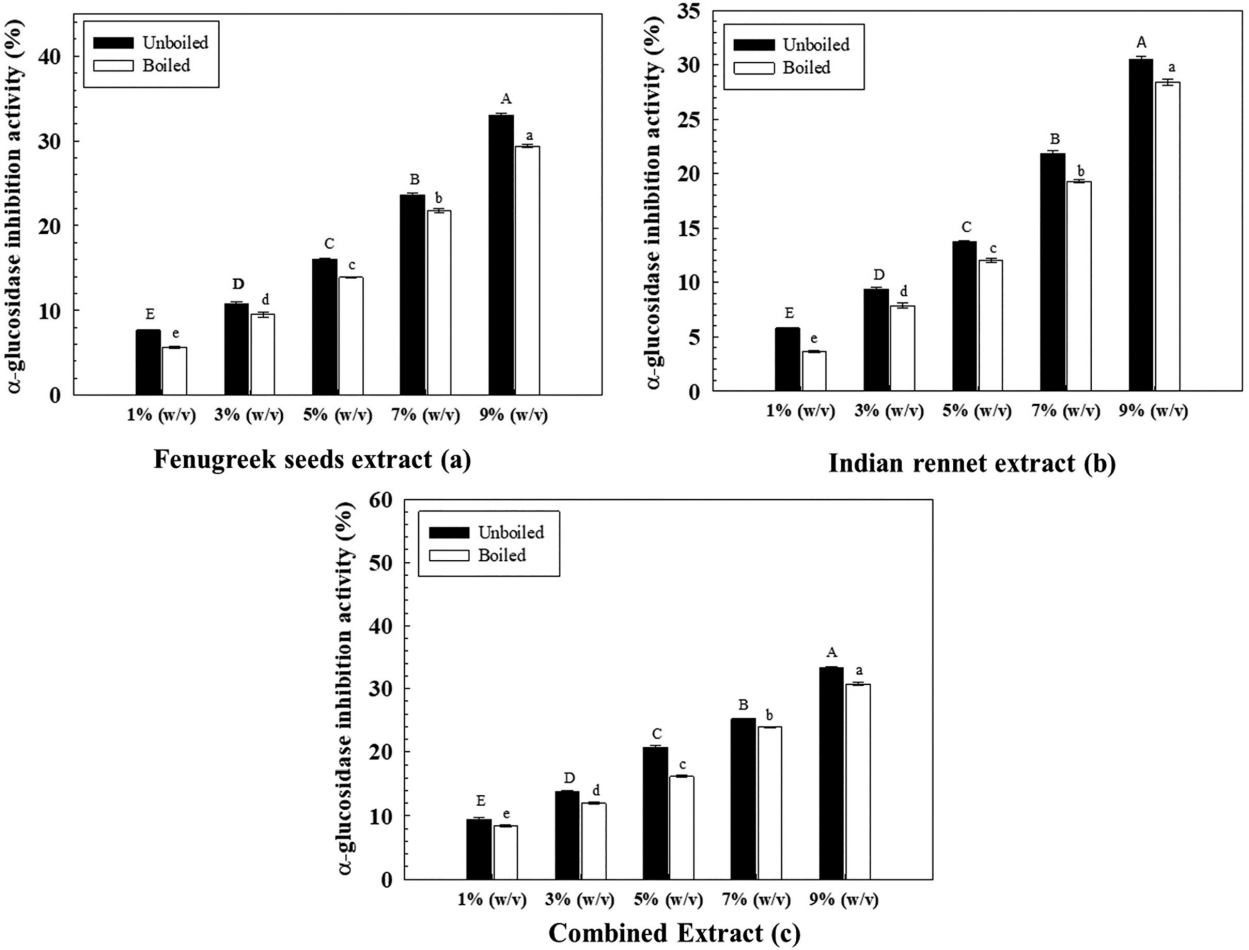
α‐glucosidase activity of extracts. Uppercase and lowercase letters show significant differences between unboiled and boiled extracts.

For fenugreek seed extract (Figure 6a), the highest inhibitory activity against α‐glucosidase (33.1%) was shown by the 9% (w/v) unboiled FS extract and the lowest activity in 1% extract (7.6%). The IC_50_ values for unboiled and boiled fenugreek seeds were measured as 14.97% and 16.39%. While IR extract also exhibited the same linear trend as the maximum concentration, i.e., 9% showed the highest inhibitory potential (30.470 ± 0.651%) (Figure 6b) with the IC_50_ values as 15.92% and 16.10% for unboiled and boiled IR extracts.

The combined extract showed a synergistic effect, with the highest inhibition activity (Figure 6c) recorded in 9% extract and lowest in 1% extract in a dose‐dependent manner. The IC_50_ values were recorded as 14.98% and 16.24% for unboiled and boiled combined extracts, respectively.

Various studies tested herbs for their α‐glucosidase inhibition potential. A study conducted by Prithiksha et al. (2022) tested the α‐glucosidase activity of aqueous fenugreek seed extract, and the results indicated that α‐glucosidase inhibition activity of extracts (100–500 μg/mL) ranged from 21% to 58% linearly. Moreover, Ganeshpurkar et al. (2013) also measured α‐glucosidase inhibition activity in methanolic and aqueous fenugreek extracts, and the results depicted that the aqueous extract showed maximum inhibition activity of 32% at the highest concentration (250 μg/mL), while the methanolic extract showed 53% inhibition activity at the same concentration. The results concluded that aqueous extracts showed lower α‐glucosidase activity than organic solvents. Meeran et al. (2020) tested Indian rennet extract at different concentrations ranging from 100 to 500 μg/mL in methanol as the solvent. The % inhibition activity ranged from 0% to 45.4% linearly, with the IC_50_ value of 314.3 μg/mL. However, there is no comparable literature available on aqueous Indian rennet extracts.

### 3.5 Sensory evaluation of pulao

The mean ranks regarding the sensorial parameters were tested using the Kruskal–Wallis test, and the results showed significant variation among all six treatments (*p* = .000). Regarding color, T_o_ was assigned the highest score, followed by T_1_ and T_2_. In comparison, T_3_–T_5_ were assigned relatively lower scores. The higher color score for T_o_–T_2_ treatments can be attributed to minimum to no change in the color of actual chickpea pulao due to the addition of herb extracts (% w/v). Fenugreek seeds usually do not show prominent color changes. Still, adding IR extracts significantly modified the pulao color, possibly due to the flowers' natural greenish to dull yellow color that turns brownish after boiling/cooking.

The mean ranks of sensory parameters such as texture and aroma scores depicted that T_o_–T_3_ were assigned higher scores as adding herb extracts did not impact the product's texture compared to T_4_ and T_5_, resulting in relatively lower scores. This might be attributed to the inability to chew rice due to the bitter aftertaste owing to the addition of a relatively higher concentration of Indian rennet herb extracts (Khan et al., 2021).

**FIGURE 7:**
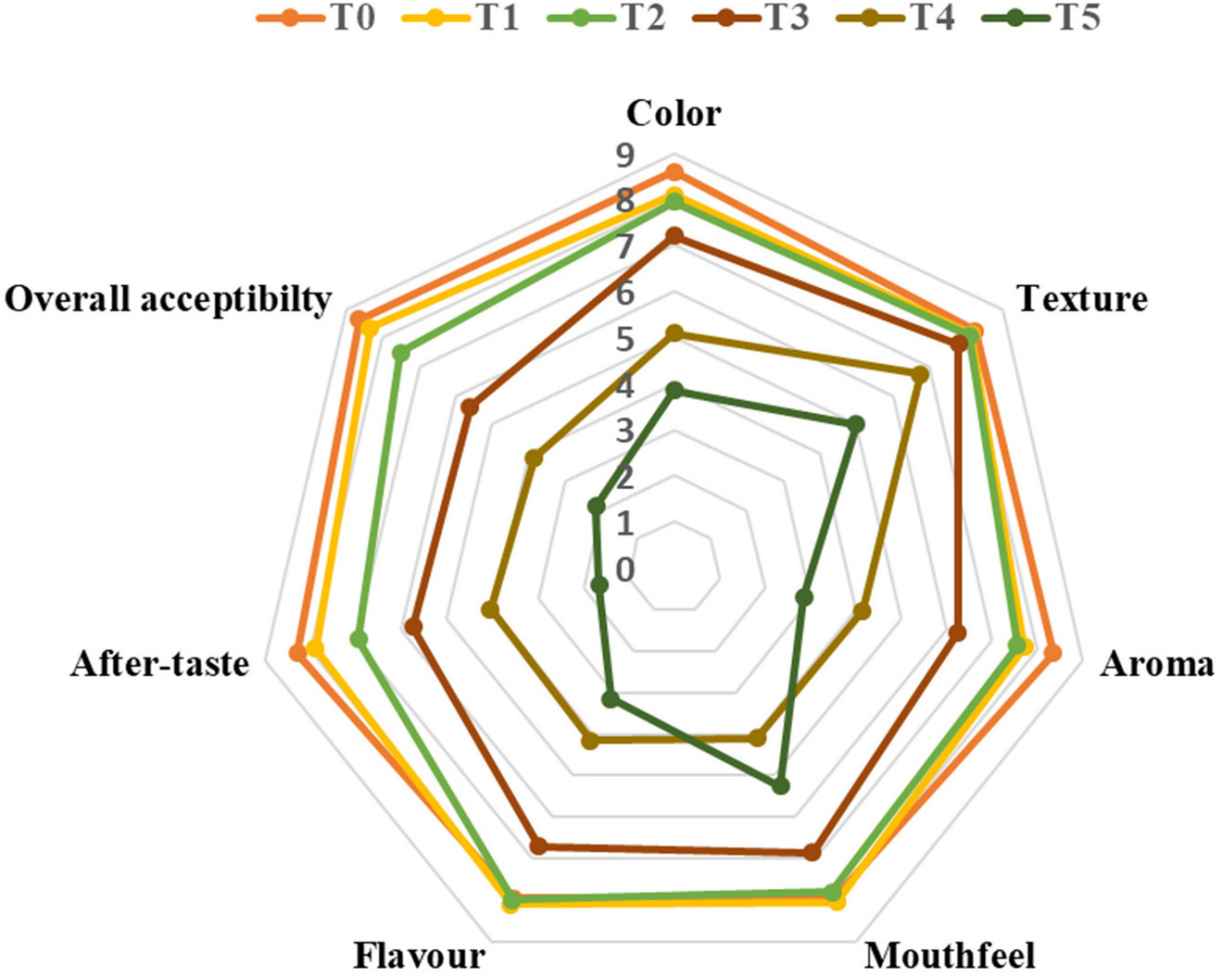
Sensory evaluation of chickpea pulao.

Similarly, the highest scores for aftertaste were assigned to T_o_–T_2_ due to lower concentrations of herb extracts. In contrast, as the concentration increased, the scores assigned to the treatments with higher concentrations decreased. The mean scores of overall acceptability (Figure 7) showed that T_o_–T_2_ was preferred more than T_3_–T_5_. Overall, as the concentration of herb extracts increased, the acceptability scores decreased. Participants' feedback revealed that higher concentrations resulted in a somewhat bitter aftertaste due to higher concentrations of Indian rennet, which has a bitter aftertaste and unappealing aroma. The T_2_ (3% FS and IR extracts) was deemed acceptable to be selected for the in vivo trial in diabetic participants.

### 3.6 Human trials

#### 3.6.1 Participants' demographics and dietary history

A total of 12 participants (6 men and 6 women) with type 2 diabetes with a mean age of 53.33 ± 4.77 years, mean BMI; 31.34 ± 2.86 kg/m^2^, mean waist circumference; 42.49 ± 3.09 participated in this study trial. With regard to the subject's medical history, the mean random blood sugar level (250.83 ± 34.23 mg/dL), mean fasting blood sugar levels (200.83 ± 28.67 mg/dL), mean cholesterol levels (268.92 ± 44.07 mg/dL), and mean HbA1C levels (7.63 ± 0.61%) were recorded before the start of the trial. Family history revealed that all 12 participants had a family history of diabetes, whereas 11 out of 12 had a family history of hypertension, and 4 out of 12 had a family history of CVD. Recruited participants had no underlying disease or any allergies.

Participants were asked about the frequency of consumption of different food groups based on a 7‐point scale. All questions regarding the food frequency questionnaire were grouped into eight major food groups: grains, meat, dairy, fruits, vegetables, fried food items (fats and oils), bakery items, and added sugars. The mean scores of all food groups of recruited participants are mentioned in Table 2.

**TABLE 2:** Mean score of dietary history of recruited participants.

Average consumption of food groups	Mean scores	Average consumption of food groups	Mean scores
Grains	2.542 ± 0.865	Vegetables	2.520 ± 0.516
Meat	4.082 ± 0.923	Fried Food items	4.5416 ± 1.339
Dairy	2.417 ± 0.655	Bakery Items	3.458 ± 1.195
Fruits	3.251 ± 1.713	Added sugars	3.551 ± 0.832

#### 3.6.2 Study design

The mean duration of the study trial was twenty‐one days, with 7 days of washover time in between. Moreover, participants' regular medicine and insulin dosage were not changed during the trial period. Furthermore, none of the subjects were told to alter their daily routine during this study period. The participants were instructed not to skip a single day of this trial, to follow the timings of consuming the provided product, and to measure blood sugar levels beforehand. In addition, none of the subjects became hypoglycemic during the study trial.

#### 3.6.3 Outcome measures

##### Mean blood sugar level on different days

**FIGURE 8:**
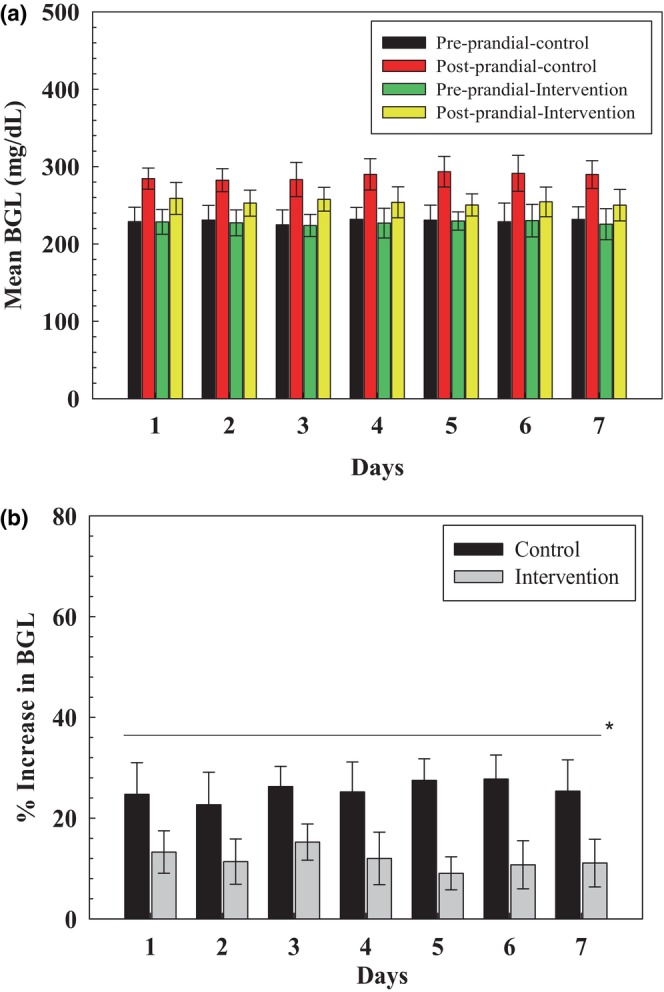
The graphical representation of (a) mean blood sugar level variations at different days. (b) Comparison between the % increase in postprandial blood sugar levels. *Significant level at 99% probability level.

The mean values of preprandial and postprandial blood sugar levels on all days were taken for statistical analysis to compare the change in participants' blood sugar levels for days. The paired comparison between preprandial and postprandial blood sugar levels after consuming the control recipe was significant (*p* = .000), which describes that there was a significant increase in postprandial blood glucose levels after consuming the control recipe as recorded the same when preprandial and postprandial blood sugar levels (Figure 8a) after consuming intervention recipe were compared (*p* = .000).

On the other hand, the difference between postprandial blood glucose levels after consuming the control recipe and intervention recipe was significant (*p* = .000), clearly depicting that consumption of the intervention recipe led to a decrease in blood glucose levels of the recruited subjects when compared daywise.

To further measure the extent of the increase in blood sugar levels, the % increase in blood sugar levels after consuming control as well as intervention recipe was calculated, and results showed that mean increase (Figure 8b) in BGL of participants was recorded as 25.61 ± 5.41 and 11.80 ± 4.32 mg/dL after consuming control recipe and intervention recipe, respectively.

##### Mean blood sugar level of all participants

**FIGURE 9:**
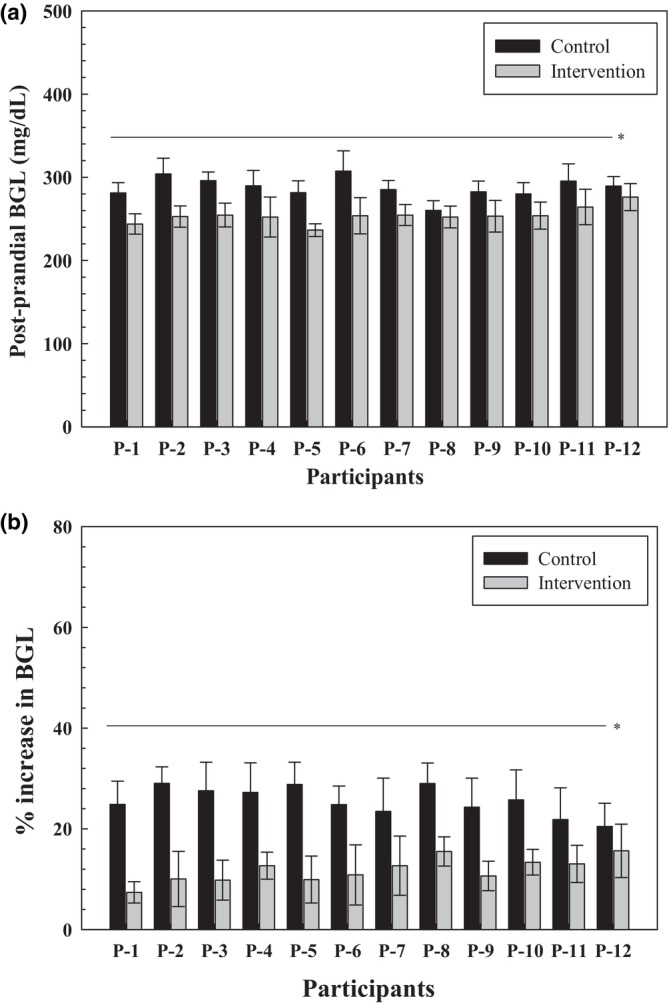
(a) Comparison between postprandial blood sugar levels. (b) % increase in blood sugar levels. *Significant level at 99% probability level.

The mean blood sugar levels of all 12 participants were also compared. A highly significant difference (Figure 9a) was measured between postprandial blood sugar levels after consuming both control and intervention recipes (*p* = .000). To further clarify the actual increase in blood sugar levels after consuming the control and intervention recipes (Figure 9b), the % increase in blood sugar levels was calculated. The paired *t*‐test showed that the control recipe resulted in higher postprandial blood sugar levels than the intervention recipe.

Many studies have conducted in vivo trials to determine the antidiabetic potential of these herbs. Ranade and Mudgalkar (2017) performed a study in which 60 people with type 2 diabetes were randomly assigned to receive either 10 g of fenugreek seeds soaked in hot water for 8 weeks or a placebo. The study concluded that the group receiving FS had significantly improved fasting blood sugar levels, glycated hemoglobin (HbA1c), and insulin resistance compared to the placebo. In another study, Gaddam et al. (2015) carried out a 3‐year randomized, controlled, and parallel study to investigate the effects of 5 g of fenugreek seed powder twice daily in patients with type 2 diabetes. The study found a significant reduction in plasma blood glucose, postprandial blood glucose levels, and LDL, with a significant increase in plasma insulin levels observed in the treatment group.

Similarly, another study, Geberemeskel et al. (2019), investigated the impact of fenugreek powder. 114 newly diagnosed type 2 diabetic individuals were chosen and randomly assigned to two groups. The intervention group received 25 g of fenugreek seed powder orally twice daily for 1 month. The results indicated that the treatment group showed a significant decrease in total cholesterol levels and LDL, whereas a significant increase in HDL in diabetic individuals was observed as compared to the control group.

Hemalatha et al. (2004) performed trials in diabetic rats; Indian rennet extract was administered at 1 g/kg. Results showed that blood glucose levels were significantly reduced in diabetic rats. Similarly, in another study conducted in streptozotocin‐induced diabetic rats, an effective antidiabetic action of the aqueous extract of Indian rennet dried fruits was shown at a dosage of 1 g/kg body weight, without any obvious adverse effects (Jaiswal et al., 2009). Datta et al. (2013) also conducted a study on rat models and determined the effectiveness of Indian rennet‐dried extract in experimental rats. Results concluded that it could be used as an adjunct therapy to treat hyperglycemia and diabetes.

### 3.7 Conclusion

The use of traditional herbs like fenugreek seeds and Indian rennet flowers has been under research to use as medicinal plants since prehistoric times. Much research is being done to analyze selected herbs' antioxidant and antidiabetic potential. The analysis of this study revealed that the herbs chosen showed good antioxidant and antidiabetic potential in a dose‐dependent manner. Boiled extracts showed a significant decrease in DPPH activity, α‐amylase, and α‐glucosidase inhibition activity compared to raw unboiled extracts mainly due to heat‐labile compounds and a significant increase in total phenolic contents after boiling as phenolics are mostly organic compounds, and they survive higher temperatures much easier as compared to inorganic constituents. However, the 21‐day single‐blind placebo‐controlled cross‐over design showed that the intervention recipe led to a significant decrease (*p* = .000) in postprandial hyperglycemia in recruited participants with respect to days. In summary, the addition of traditional herbs to local cuisine may have a beneficial impact on the management of diabetes.

## AUTHOR CONTRIBUTIONS

**Misha Arooj:** Data curation (equal); formal analysis (equal); investigation (equal); methodology (equal); visualization (equal); writing – original draft (equal). **Zaheer Ahmed:** Methodology (equal); validation (equal); visualization (equal); writing – original draft (equal). **Nauman Khalid:** Conceptualization (equal); funding acquisition (equal); methodology (equal); project administration (equal); validation (equal); writing – review and editing (equal). **Hafiz A. R. Suleria:** Funding acquisition (equal); validation (equal); writing – review and editing (equal).

## FUNDING INFORMATION

The authors did not receive any funding for conducting this research.

## CONFLICT OF INTEREST STATEMENT

The authors declare no conflict of interest in writing this research article.

## ETHICS STATEMENT

The ethical approval to conduct this study was obtained from the bioresearch ethical committee of the University of Management and Technology Lahore via. Approval number UMT/IRB/PostGrad/Res/2022‐03‐R002‐C‐1. The clinical trial was listed on ClinicalTrial.gov (NCT06095622). Moreover, the study was conducted following the Declaration of Helsinki Protocol principles.

## Data Availability

The data is available by requesting from corresponding authors.
